# CYP3A4 and VDR gene polymorphisms and the risk of prostate cancer in men with benign prostate hyperplasia

**DOI:** 10.1038/sj.bjc.6600825

**Published:** 2003-03-18

**Authors:** M T Tayeb, C Clark, N E Haites, L Sharp, G I Murray, H L McLeod

**Affiliations:** 1 Department of Medicine and Therapeutics, Institute of Medical Sciences, University of Aberdeen, Foresterhill, Aberdeen, UK; 2 Department of Molecular and Cell Biology, Institute of Medical Sciences, University of Aberdeen, Foresterhill, Aberdeen, UK; 3 Department of Pathology, Institute of Medical Sciences, University of Aberdeen, Foresterhill, Aberdeen, UK; 4 Department of Medical Sciences, Faculty of Medicine, Umm Al-Qura University, Makkah, Saudi Arabia; 5 Department of Medicine, Washington University, St Louis, USA

**Keywords:** CYP3A4, vitamin D receptor, prostate cancer, polymorphism, benign prostate hyperplasia, gene–gene interaction

## Abstract

Prostate cancer (PRCa) is one of the most common causes of cancer death in men and determinants of PRCa risk remain largely unidentified. Benign prostatic hyperplasia (BPH) is found in the majority of ageing men and has been associated with PRCa. Many candidate genes have been suggested to be involved in PRCa, such as those that are central to cellular growth and differentiation in the prostate gland. The vitamin D receptor (*VDR*) and *CYP3A4* have been shown to be involved in the regulation of cell proliferation and differentiation in prostate cells. Genetic variations of these genes have been associated with PRCa in case–control studies and may be useful to detect BPH patients that have a higher risk of developing PRCa. The association between *CYP3A4* and *VDR* *Taq*I SNPs and the risk of developing PRCa have been investigated in this study by determining the variant genotype frequencies of both SNPs in 400 patients with BPH who have been followed clinically for a median of 11 years. The results of this study showed that the incidence rate of PRCa was higher in BPH patients having *CYP3A4* variant genotype compared to those with wild type (relative risk (RR)=2.7; 95% CI=0.77–7.66). No association between variant genotype and risk of developing PRCa was observed with the *VDR* *Taq*I variant genotype. In addition, the results of combined genotype analysis of these two SNPs showed a borderline significant association between *CYP3A4* and *VDR* *Taq*I combined variant genotypes and PRCa risk (RR=3.43; 95% CI=0.99–11.77). While independent confirmation is required in further studies, these results provide a potential tool to assist prediction strategies for this important disease.

Prostate cancer (PRCa) constitutes a major health issue worldwide. Prostate cancer is one of the most common causes of cancer death in men (Parker *et al*, 1996; Prior and Waxman, 2000). The incidence of PRCa increases with age and it is estimated that 80% of men would be affected by the age of 80 years (Holund, 1980). The aetiology of PRCa is unclear, although current evidence suggests that PRCa is the result of multiple factors that include ethnicity, environmental, genetics, hormonal and dietary factors (Pienta and Esper, 1993; Whittemore *et al*, 1995; Wingo *et al*, 1996; Hsieh *et al*, 1999; Tzonou *et al*, 1999; Lichtenstein *et al*, 2000). Benign prostatic hyperplasia (BPH) is a non-neoplastic enlargement of the prostate. Benign prostatic hyperplasia is extremely common, with a rapid increase in prevalence in the fourth decade of life. According to epidemiological studies, most cancers are associated with BPH elsewhere in the prostate (83.3%; Carter and Coffey, 1990; Bostwick *et al*, 1992) and approximately 3–20% of patients who have undergone transurethral prostatectomy (TURP) or open prostatectomy for BPH subsequently develop PRCa (Armenian *et al*, 1974; Schwartz *et al*, 1986; Bostwick *et al*, 1992). Compared to men without BPH, those with the condition have a five-fold raised risk of developing PRCa and a four-fold raised risk of death from PRCa (Armenian *et al*, 1974). A previous study reported that a family history of prostate disease (PRCa or BPH) was more frequently seen in relatives of men with BPH (20%) than in relatives of men with PRCa (12.8%) or in healthy controls (5.1%) (Schuman *et al*, 1977). In addition, *in vitro* malignant transformation of BPH tissue has been previously reported (Chen and Heidelberger, 1969; Fraley *et al*, 1970; Frank and Wilson, 1970). These results suggest that common genetic mechanisms may predispose to benign and malignant prostate disease. Moreover, these results suggest that BPH may be part of a premalignant environmental condition in the prostate gland. With the increasing incidence of PRCa in many populations, there is an urgent need for the identification of molecular markers that can serve as indicators of disease risk to focus chemoprevention and early detection strategies. Many candidate PRCa genes have been suggested, including genes influencing cellular growth and differentiation. The cytochrome *P*450 3A4 enzyme (CYP3A4) is a member of the human *P*450 family. CYP3A4 protein is responsible for hydroxylation of testosterone, which results in the deactivation of the hormone function (Waxman *et al*, 1988; Yamazaki and Shimada, 1996). A single-nucleotide polymorphism (SNP) in the *CYP3A4* promoter (−290 A to G) was previously reported with two *CYP3A4* alleles; CYP3A4^*^1A is the wild type (−290A) and *CYP3A4-V*, now designated CYP3A4^*^1B (−290G), is the variant (Rebbeck *et al*, 1998; Sata *et al*, 2000). The functional influence of this SNP is unclear, but initial *in vitro* and *in vivo* studies suggest a role in transcriptional control leading to altered CYP3A4 enzyme activity for a number of substrates, including testosterone (Amirimani *et al*, 1999; Rebbeck, 2000; Wandel *et al*, 2000). Genetic epidemiology studies found that the CYP3A4^*^1B allele was associated with higher clinical stage and grade of PRCa (Rebbeck *et al*, 1998; Paris *et al*, 1999; Kittles *et al*, 2002). The CYP3A4^*^1B allele frequency has been shown in various studies to vary markedly between ethnic groups and match solely with the incidence of PRCa based on ethnicity (Walker *et al*, 1998; Ball *et al*, 1999; Paris *et al*, 1999; Sata *et al*, 2000; Tayeb *et al*, 2000; Kittles *et al*, 2002). The highest incidence of PRCa was found in African Americans, intermediate in Caucasians, and the lowest in Asians (Pienta and Esper, 1993; Wingo *et al*, 1996). Vitamin D has been implicated in PRCa, with several epidemiological studies linking low vitamin D levels with increased risk of PRCa (Schwartz and Hylka, 1990; Corder *et al*, 1993). Calcitriol, the biologically active metabolite of vitamin D, 1,25-dihydroxyvitamin D_3_, has been shown to inhibit prostate cell growth (Skowronski *et al*, 1993; Hedlund *et al*, 1996). The action of vitamin D is mediated through binding to its nuclear receptor (VDR). The inherited *Taq*I SNP in exon 9 of the *VDR* 3′UTR regions (C352T) has been demonstrated to affect vitamin D levels (Morrison *et al*, 1994; Ma *et al*, 1998). Previous studies observed an association between the *Taq*I SNP and PRCa risk (Taylor *et al*, 1996; Correa-Cerro *et al*, 1999; Hamasaki *et al*, 2001). Our previous nested case–control association studies found that the frequencies of the CYP3A4^*^1B and VDR *Taq*I TT genotypes are higher among BPH patients who subsequently develop PRCa than among BPH control patients (Odds ratio, OR: 5.2 and 5.16, respectively; Tayeb *et al*, 2002 submitted). Moreover, we found that the frequency of CYP3A4^*^1B and *VDR* TT combined genotypes is increased in BPH patients who developed PRCa later on in their life compared with BPH patients who did not, and the risk of developing PRCa was 13-fold higher in BPH patients having the CYP3A4^*^1B and VDR TT combined genotypes than the control (Tayeb *et al*, 2002, submitted). The association between *CYP3A4* and *VDR* *Taq*I SNPs, and the risk of developing PRCa in BPH patients have been investigated further in this study by determining the CYP3A4^*^1B and VDR TT genotype frequencies in 400 patients with BPH who have been followed up to 11 years.

## MATERIALS AND METHODS

Data for BPH patients from years 1989 to 1990 (Northeast Scotland; Grampian region) were collected using the University of Aberdeen Department of Pathology data bases. In total, 1010 samples were identified. Data for PRCa from years 1989 to April 2000 were also collected and 44 of the 1010 BPH patients (4.4%) subsequently developed PRCa in the period 1989–April 2000. The geographic region has very little population migration over generations and is served by a single pathology department. Of the 1010 BPH samples, 400 were randomly selected for further molecular analysis, of which 21 had subsequently developed PRCa (5%). All sections were rereviewed by pathologist to confirm the diagnosis.

### Genotyping

DNA was extracted from formalin-fixed, paraffin-embedded tissues. The tissue sections were deparaffinised with xylene and ethanol, and then DNA was isolated by proteinase K digestion (Frank *et al*, 1996). A 289 bp fragment CYP3A4^*^1B was amplified by PCR and screened using single-strand conformation polymorphism (SSCP) analysis (Tayeb *et al*, 2000). Previously described primer set was used to amplify the region of 198 bp around the *VDR* *Taq*I polymorphic region (Lundin *et al*, 1999). Genomic DNA (100–500 ng) was subjected to PCR amplification in a 25 *μ*l reaction mixture containing 10 × PCR buffer (MBI, Sunderland, UK), 1 mM MgCl_2_ (MBI), 200 *μ*M dNTP mix (Bioline, London, UK), 10 pmol of each primer, 1 U of *Taq* polymerase (Roche, Lewes, UK), and sterilised distilled water. The genomic DNA was initially denatured at 94°C for 2 min and thereafter subjected to 35 cycles of PCR amplification with denaturation for 1 min at 94°C, annealing for 2 min at 60°C, extension for 2 min 30 s at 72°C, and a final extension step at 72°C for 10 min. The PCR products were digested with the *Taq*I restriction endonuclease (Roche, Lewes, UK) at 65°C for 5 h. Genotypes for the SNPs were determined after separation on a 3% agarose gel. Individuals were scored as TT homozygous (absence of *Taq*I restriction sites), Tt heterozygotes, or tt (presence of *Taq*I restriction sites).

### Statistical analysis

Random selection for cohort samples was made using Minitab software version 12.1. The incidence rate and the relative risk (RR) of developing PRCa with studied markers and the power of the cohort study were calculated using Stata 1.0 software.

## RESULTS

### CYP3A4^*^1B frequencies across populations

Our cohort study had 83% power to detect an RR of 4. The overall incidence rate of PRCa in this study was 645 per 100 000 men-year. Genotype frequencies of the *CYP3A4* SNP in the cohort population are shown in Table 1

**Table 1:** Distribution of *CYP3A4* genotype frequencies in the cohort population

		**Genotype frequency (%)**		
**Population**	***n***	**A/A**	**A/G**	**G/G**
Benign prostatic hyperplasia patients developing PRCa during follow-up period	21	16 (76)	4 (19)	1 (5)
Benign prostatic hyperplasia patients not developing PRCa during follow-up period	379	344 (91)	35 (9)	0 (0)

*n*=number of subjects, A/A (homozygote wild type; CYP3A4^*^1A/CYP3A4/1A), A/G (heterozygote; CYP3A4^*^1A/CYP3A4^*^1B) and G/G (homozygote variant; CYP3A4^*^1B/CYP3A4^*^1B).

. From Table 1, the frequencies of the CYP3A4^*^1B homozygote and heterozygote genotypes were higher in BPH patients who developed PRCa during the time of follow-up compared to BPH patients who did not. Genotype frequencies of *CYP3A4* SNP and incidence rate of PRCa in the cohort study are shown in Table 2

**Table 2:** Distribution of *CYP3A4* genotype frequencies and incidence rate of PRCa in the cohort population

	**CYP3A4 SNP genotypes**	
	**A/A**	**A/G and G/G**
Benign prostatic hyperplasia patient who developed PRCa during follow-up	16 (76%)	5 (24%)
Person-years of follow-up	2918	340
Incidence rate of PRCa per 100 000 men-year	548	1471

. From Table 2, the incidence rate of PRCa was higher in BPH patients with CYP3A4^*^1B genotype compared to BPH patients with CYP3A4^*^1A homozygotes. The RR of developing PRCa was 2.7 (95% CI=0.77–7.66) in BPH patients having a CYP3A4^*^1B genotype.

### *VDR* TT genotype frequency across population

The power of the cohort study was predicted to detect an RR of 4 with 82% power. Genotype frequencies of the *Taq*I SNP in the cohort population are shown in Table 3

**Table 3:** Distribution of *Taq*I genotype frequencies in the cohort population

		**Genotype frequency (%)**		
**Population**	***n***	**TT**	**Tt**	**tt**
Benign prostatic hyperplasia patient developed PRCa during follow-up	21	7 (33)	10 (48)	4 (19)
Benign prostatic hyperplasia patient who did not develop PRCa	379	136 (36)	181 (48)	62 (16)

*n*=number of subjects.

. From Table 3, the frequency of the TT genotype is similar in BPH patients who developed PRCa to BPH patients who did not (33 and 36%, respectively). The incidence rate of PRCa was lower in BPH patients with TT genotype compared to BPH patients with Tt or tt genotypes (Table 4

**Table 4:** Distribution of *Taq*I genotype frequencies and incidence rate of PRCa in the cohort population

	**TaqI SNP genotypes**	
	**TT**	**Tt and tt**
Benign prostatic hyperplasia patient who developed PRCa during follow-up	7 (33%)	14 (67%)
Person-years of follow-up	1198	2060
Incidence rate of PRCa per 100 000 men-years	584	680

). The RR of developing PRCa was 0.86 (95% CI=0.29–2.28) in BPH patients having a TT genotype. However, the results were not statistically significant.

### Combined genotype analysis

The frequencies of the CYP3A4^*^1B homozygote, heterozygote (A/G and G/G), and *VDR* TT combined genotypes were higher in BPH patients who developed PRCa during the time of follow-up compared to BPH patients who did not (Table 5

**Table 5:** Distribution of *CYP3A4* and *VDR Taq*I combined genotype frequencies in the cohort population

		**Genotype frequency (%)**	
**Population**	***n***	**A/G G/G and TT**	**Other**
Benign prostatic hyperplasia patients developing PRCa during follow-up period	21	3 (14)	18 (86)
Benign prostatic hyperplasia patients not developing PRCa during follow-up period	379	13 (3)	366 (97)

*n*=number of subjects, other=other combined genotypes.

). The incidence rate of PRCa was higher in BPH patients with CYP3A4^*^1B (A/G and G/G) and *VDR* TT combined genotypes compared to BPH patients with other combined genotypes (Table 6

**Table 6:** Distribution of *CYP3A4* and *VDR Taq*I genotype frequencies and incidence rate of PRCa in the cohort population

	**CYP3A4&VDR TaqI SNPs genotypes (%)**	
	**A/G, G/G and TT**	**Other**
Benign prostatic hyperplasia patient who developed PRCa during follow-up	3 (14)	18 (86)
Person-years of follow-up	151	3107
Incidence rate of PRCa per 100 000 men-years	1987	579

Other=other combined genotypes.

). The RR of developing PRCa was 3.43 (95% CI=0.99–11.77) in BPH patients having a CYP3A4^*^1B and *VDR* TT combined genotypes.

## DISCUSSIONS

Both PRCa and BPH are common diseases for which the incidence increases with age. Previous studies have defined a significant association between BPH and developing PRCa (Armenian *et al*, 1974; Bostwick *et al*, 1992). With the increasing incidence of BPH in the ageing population, there is an urgent need for the identification of molecular markers that can serve as prognostic indicators for developing PRCa in those patients with BPH. Germline and somatic variations in genes directly involved in the regulation of prostate cell growth might be critically important in understanding the carcinogenesis of PRCa, as these variants might be used as diagnostic, prevention, and prognostic markers for PRCa. The primary aim of this study was to identify molecular markers that are important in the development of PRCa in patients with BPH. If molecular markers in patients with BPH are shown to be predictors for eventually developing PRCa, then more intensive surveillance and/or early treatment could be offered to those selected patients. Such an approach is also likely to lead to improvements in survival. In the converse situation, those patients who do not have a high risk of developing PRCa could be offered standard follow-up monitoring. Our previous nested case–control association studies showed that a constitutive *CYP3A4* and *VDR* *Taq*I SNPs are associated with a group of men with BPH who are at an increased risk of PRCa (Tayeb *et al*, 2002, submitted). The association between these two SNPs and risk of developing PRCa have been investigated further in this study by determining the CYP3A4^*^1B and *VDR* TT genotype frequencies in 400 patients with BPH (1989–1990). The median years of follow-up for these patients were 11 years and during the time of follow-up, 21 BPH patients developed PRCa. The results of this study showed that the incidence rate of PRCa was higher in BPH patients having a CYP3A4^*^1B genotype compared to those homozygous for CYP3A4^*^1A, but it was not statistically significant. The RR of BPH patients developing PRCa was 2.7, although results failed to reach significance at the 5% level. Regarding *VDR Taq*I SNP, the RR of BPH patients developing PRCa was 0.86 in patients with the TT genotype, although results were not statistically significant. This lack of significance could be because of the limited power of the cohort study, as the power of the study was determined to detect an RR of 4 with 83 and 82% for *CYP3A4* and *VDR Taq*I SNPs, respectively. The power of a cohort study depends on several factors: (1) the number of subjects enrolled in the cohort, (2) the time for which each subject is followed-up, (3) the rate at which events (PRCa) occur in the cohort, (4) the frequency of ‘exposure’ to the hypothesised risk factor in the cohort (in this case, the frequency of the *CYP3A4* genotypes or/and *VDR* *Taq*I genotypes, which had been hypothesised to be associated with increased risk of developing PRCa), (5) the size of RR which the investigator want to detect. As these factors change, the power of the study, or the necessary sample size, also changes. PRCa is likely to be caused by complex interactions between genetic, endocrine, and environmental factors. Ethnic differences in the risk of developing PRCa suggest that in addition to environmental factors, common genetic variants with low penetrance and high population attributable risk may play an essential role in the aetiology of PRCa. This study examined the data for gene–gene interactions between putative risk genotypes, CYP3A4^*^1B and *VDR* TT. These genetic variations confer an increased risk for the development of PRCa through their mediation of prostate cell growth and differentiation. The identification of evidence of a significant interaction (patients with both risk genotypes) may not necessarily indicate that the two genes are synergistic. They may instead influence risk via independent mechanisms. Gene–gene interactions might be important for the development of PRCa and this interaction needs to be explored. The results of this study showed that BPH patients who subsequently developed PRCa have significantly different frequency of harbouring *CYP3A4*, and *VDR* at risk genotypes than those BPH patients who did not develop PRCa (13-fold, Tayeb *et al*, 2002, submitted). It is interesting to notice that the ORs obtained from these combined genotypes (A/G and TT) were higher than those ORs obtained from each individual variant: 5.2 and 5.16 for heterozygous CYP3A4^*^1B and homozygous *VDR* TT, respectively (Tayeb *et al*, 2002, submitted). This study observed a borderline significant association between these combined genotypes and PRCa risk (RR=3.43; 95% CI=0.999–11.770). The RRs for these combined genotypes were higher than those obtained for each individual marker: 2.7 and 0.86 for heterozygous and homozygous CYP3A4^*^1B, and homozygous *VDR* TT, respectively. Calcitriol has been reported to inhibit PRCa proliferation and to promote a more differentiated phenotype. *VDR*, *Taq*I SNP has been demonstrated to affect transcriptional activity and mRNA stability, thus altering the abundance of VDR, and in turn affects vitamin D level (Morrison *et al*, 1994). In addition, higher levels of calcitriol have been reported in those who are homozygous for the t (*Taq*I site) allele relative to those who are homozygous for the T (no site) allele (Morrison *et al*, 1994; Ma *et al*, 1998). It has been speculated that men with the CYP3A4^*^1B genotype may have altered testosterone metabolism, promoting androgen-mediated prostate carcinogenesis and the occurrence of PRCa (Rebbeck *et al*, 1998). It might be that BPH patients harbouring both *CYP3A4* and *VDR* *Taq*I combined risk genotypes have a higher level of androgen hormones and lower level of calcitriol, which might lead to an increase in prostate cell growth and reduce the level of differentiation and apoptosis, which might result in the occurrence of PRCa. However, larger studies are clearly needed to confirm these assumptions. If confirmed, the genetic risk factors examined in this study (*VDR*, and *CYP3A4*) are among the strongest risk factors yet identified for PRCa. The finding of this study is consistent with a multigenic model of PRCa, where PRCa risk is influenced by gene–gene and gene–environment interactions. On the basis of the joint effect of several loci, one might ultimately be able to construct a risk profile that could predict the development of the disease and could allow for a more meaningful decision making regarding optimal treatment strategies.
